# Synthesis of CNTs/CoNiFe-LDH Nanocomposite with High Specific Surface Area for Asymmetric Supercapacitor

**DOI:** 10.3390/nano11092155

**Published:** 2021-08-24

**Authors:** Jianwei Wang, Qian Ding, Caihui Bai, Feifei Wang, Shiguo Sun, Yongqian Xu, Hongjuan Li

**Affiliations:** Shaanxi Key Laboratory of Natural Products and Chemical Biology, College of Chemistry and Pharmacy, Northwest A&F University, Xianyang 712100, China; wangjw19980228@163.com (J.W.); qianding@nwafu.edu.cn (Q.D.); baicaihui@nwafu.edu.cn (C.B.); wangfeifei6586@163.com (F.W.); sunsg@nwsuaf.edu.cn (S.S.); xuyq@nwsuaf.edu.cn (Y.X.)

**Keywords:** layered double hydroxide, carbon nanotubes, nanocomposite, electrode materials, asymmetric supercapacitor

## Abstract

Ternary layered double hydroxide (LDH) materials have shown promising application in hybrid supercapacitors. However, the low electrical conductivity of LDHs is still a restriction to their performance. Herein, carbon nanotubes/cobalt–nickel–iron LDH (CNTs/CoNiFe-LDH) hybrid material was prepared by a one-step hydrothermal approach for the first time. The presence of CNTs improved the conductivity and surface area of the electrode, leading to an enhanced electrochemical performance. The CNTs/CoNiFe-LDH hybrid electrode exhibited high specific capacity 170.6 mAh g^−1^ at a current density of 1 A g^−1^, with a capacity retention of 75% at 10 A g^−1^. CNTs/CoNiFe-LDH//AC asymmetric supercapacitor (ASC) was also assembled, which had high specific capacitance (96.1 F g^−1^ at the current density of 1 A g^−1^), good cycling stability (85.0% after 3000 cycles at 15 A g^−1^) and high energy density (29.9 W h kg^−1^ at the power density of 750.5 W kg^−1^). Therefore, the CNTs/CoNiFe-LDH material could be used for hybrid supercapacitor electrodes.

## 1. Introduction

In recent years, the development of high-performance energy storage devices has gained significant attention [1,2,3,4,5,6]. Hybrid supercapacitors are considered as novel energy storage devices due to their fast charge–discharge rate capability, long-term cycle stability, high energy density and power density [7]. For example, Wei et al. fabricated CNT/Co_3_S_4_@NiCo LDH composites for hybrid supercapacitors [8]. In most instances, hybrid supercapacitors combine a supercapacitor-type electrode (such as carbon materials) with a battery-type electrode (such as transition metal oxides/hydroxides) into a single device [9,10,11,12,13].

Layered double hydroxide (LDH) materials, with high reversible charge/discharge abilities, structure-tunability, high specific capacity and environmental friendliness, have been extensively used as battery-type electrodes [14,15]. Nickel- or cobalt-based LDH materials have been investigated as outstanding electrodes for hybrid supercapacitors, such as CoAl-LDH [16], NiAl-LDH [17], CoNi-LDH [18,19], NiFe-LDH [20] and so on. Compared with binary LDH, ternary LDH materials, with diversified metal elements, often show excellent capacitive characteristics owning to their high active species, structural diversity and high capacity. The iron element in LDHs has been reported to be electrochemically active, which is effective in boosting up the electrochemical behavior [21,22]. Nevertheless, there is only limited research on ternary cobalt–nickel–iron LDH materials for battery-type electrodes. For example, Rohit et al. synthesized CoNiFe-LDH nanosheets with a specific capacity of 360 C g^−1^ at 0.4 A g^−1^ [21]. Li et al. fabricated FeCoNi-LDH Nanocage, which has a capacitance of 980 F g^−1^ at 1 A g^−1^ [23]. Wang et al. reported ternary LDH electrodes and studied the role of M^3+^. The capacitance of NiCoFe-LDH is 903 F g^−1^ at 1 A g^−1^ [24]. Su et al. prepared NiCoFe-LDH materials and investigated their electrochemical performance in low temperature [25]. Even though high specific capacity of NiCoFe-LDH was reported, the performances of asymmetric supercapacitor devices have not been studied. In addition, LDH materials usually have defects of low conductivity, limited specific area, unsatisfactory electrochemical stability and tendency of aggregation [26]. Many strategies have been developed to overcome this drawback, such as incorporation of heteroatoms in LDH, designing of hierarchical mesoporous structure or the formation of nanocomposites [27,28]. Hybrid electrode materials, which combine fast and reversible redox reactivity of battery-type electrode materials with the good electronic conductivity of supercapacitor electrodes, usually exhibit enhanced electrochemical characteristics [29,30]. Carbon nanotubes (CNTs), as a mature supercapacitor electrode, own superior conductivity, outstanding porous structure (with high specific area) and good electronic stability [31,32]. For instance, Tian et al. designed a high-performance wearable supercapacitor based on PANI/NCNT@CNT fibers. Porous N-CNT structure can greatly facilitate the transfer of electrons and ions [33]. Therefore, a combination of ternary LDH with reversible redox activity and conductive carbon nanotubes is expected as an effective strategy for the development of high-performance hybrid supercapacitors. To the best of our knowledge, there is no report on the hybrid electrode-based CNTs and CoNiFe-LDH.

Herein, we fabricated new CNTs/CoNiFe-LDH composites made from a supercapacitor electrode (CNTs) and a battery electrode (CoNiFe-LDH) by a one-step hydrothermal process. As expected, the CNTs/CoNiFe-LDH composite exhibited improved conductivity, higher surface area and enhanced electrochemical behavior.

## 2. Materials and Methods

### 2.1. Materials

The chemicals (CoCl_2_·6H_2_O, FeCl_3_·6H_2_O, C_6_H_5_Na_3_O_7_ and urea) were purchased from Guangdong Guanghua Sci-Tech Co., Ltd., Shantou, China. Potassium hydroxide (KOH) and NiCl_2_·6H_2_O were supplied from Guangdong Chemical Reagent Engineering-technological Research and Development Center, Shantou, China. Ethanol and HNO_3_ were purchased from Chengdu Chron Chemical Regent Co., Ltd., Chengdu, China. All chemicals were analytical reagent and used as received.

### 2.2. Preparation of Carboxylated CNTs

The pristine CNTs (10~20 nm, Shenzhen Nanotech Port Co. Ltd., Shenzhen, China.) were pretreated by dispersing CNTs in concentrated HNO_3_ (68%) under ultrasonication and refluxing for 12 h at 100 °C [34,35]. The carboxylated CNTs was obtained after filtration, washing and drying at 80 °C in vacuum.

### 2.3. Synthesis of CNTs/CoNiFe-LDH Composite

CNTs/CoNiFe-LDH composite was synthesized via a one-step hydrothermal approach. CoCl_2_·6H_2_O (94.8 mg), NiCl_2_·6H_2_O (572.2 mg), FeCl_3_·6H_2_O (216.4 mg), C_6_H_5_Na_3_O_7_ (134.5 mg) and urea (480.5 mg) were dissolved in the above-carboxylated CNTs dispersion (80 mL, 1 mg mL^−1^) by vigorous stirring. After purging with N_2_ for 5 min, the mixture was transferred into a Teflon autoclave (100 mL) and heated at 150 °C for 48 h. The product was purified by washing with ethanol and H_2_O, and dried at 80 °C in vacuum.

For comparison, CoNiFe-LDH without CNTs was also synthesized in the same way.

### 2.4. Characterization

The structure and composition of samples were examined by X-ray diffraction (XRD, Bruker D8 Advance A25, Bruker-AXS, Karlsruhe, Germany) and X-ray photoelectron spectrometer (XPS, Thermo ESCALAB 250XI, Thermo Fisher Scientific Inc., Waltham, MA, USA). Surface morphologies were observed by a field emission scanning electron microscope (FESEM, ZEISS, Sigma 300, Zeiss, Shanghai, China) and transmission electron microscopy (TEM, JEM-2100, JEOL, Tokyo, Japan). The products were also characterized by thermogravimetric analysis (TGA, SDT Q600, TA Instruments, New Castle, Delaware, USA. 10 °C min^−1^, air), N_2_ adsorption–desorption analyzer (Micromeritics ASAP 2460, Micromeritics, Atlanta, GA, USA) and Fourier-transform infrared spectroscopy (FTIR, Thermo Scientific Nicolet iS5, Thermo Fisher Scientific, Shanghai, China).

### 2.5. Electrochemical Measurements

The working electrode was fabricated by mixing active material (80 wt.%), acetylene black (10 wt.%) and polyvinylidene fluoride (PVDF, 10 wt.%) with ethanol to form a slurry, which was pressed onto nickel foam (1 cm^2^) and dried at 80 °C in vacuum. In a three-electrode system, cyclic voltammetry (CV), galvanostatic charge–discharge (GCD) and electrochemical impedance spectroscopy (EIS) were carried out on a CHI 660E electrochemical workstation in 6 M KOH, using Pt foil (4 cm^2^) and Hg/HgO electrodes as counter and reference electrodes, respectively. CNTs/CoNiFe-LDH//AC asymmetric supercapacitor (ASC) was also prepared, where CNTs/CoNiFe-LDH and activated carbon (AC) were used as positive and negative electrodes, respectively. Cycle life was conducted on a LAND CT2001A test system.

The following equations were used for calculation:

(1) $$C_{t}=\frac{I\times t}{3.6\times m}$$

(2) $$C_{S}=\frac{I\times t}{m\times ∆V}$$

(3) $$E=1/2C∆V^{2}$$

(4) $$P=\frac{E\times 3600}{t}$$

Here, Cs, Ct, E, P are specific capacitance (F g^−1^), specific capacity (mAh g^−1^), energy density (Wh kg^−1^) and power density (W kg^−1^). I, t, m, ΔV represent the constant discharge current (A), discharge time (s), mass of the active material (g) and voltage range (V). C stands for the specific capacitance of the ASC cell (F g^−1^).

## 3. Results and Discussion

**Figure 1:**
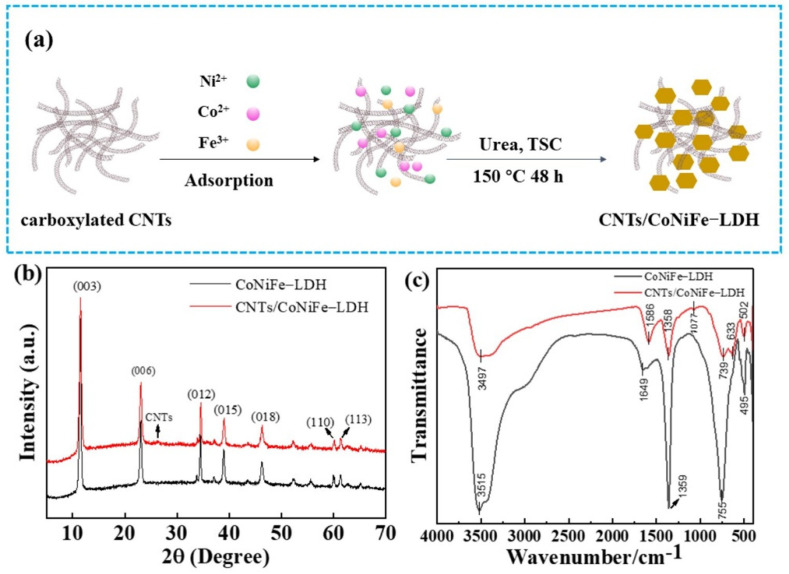
(**a**) Schematic illustration of the fabrication process of CNTs/CoNiFe-LDH composite. (**b**) XRD patterns and (**c**) FT-IR spectra of CoNiFe-LDH and CNTs/CoNiFe-LDH.

Schematic illustration of the fabrication process of CNTs/CoNiFe-LDH composite is shown in Figure 1a. Fe^3+^, Co^2+^, and Ni^2+^ ions were firstly adsorbed on the surface of negatively charged CNTs. Then, CoNiFe-LDH nanosheets were grown in situ on CNTs surface to form CNTs/CoNiFe-LDH composite under hydrothermal condition.

CoNiFe-LDH and CNTs/CoNiFe-LDH were characterized by XRD (Figure 1b). XRD curves of CoNiFe-LDH showed a series of peaks at 11.47°, 23.04°, 34.44°, 38.93°, 46.22°, 59.95° and 61.30°, which indexed to (003), (006), (012), (015), (018), (110) and (113) planes of the LDH phase [36,37]. In comparison, XRD of CNTs/CoNiFe-LDH composite showed similar peaks at 11.51°, 23.06°, 34.52°, 38.99°, 46.28°, 60.11° and 61.30°. The weak additional peak at 26.26° was attributed to (002) plane of CNTs [38,39,40], suggesting the formation of the CNTs/CoNiFe-LDH composite.

FT-IR spectra of CoNiFe-LDH and CNTs/CoNiFe-LDH were shown in Figure 1c. The broad absorption bands at about 3497 and 3515 cm^−1^ were attributed to the stretching vibrations of O−H groups and the bands at 1586 and 1649 cm^−1^ were assigned to O−H bending vibrations of interlayer H_2_O molecules [32,41]. The bands at 1358 and 1359 cm^−1^ were due to CO_3_^2−^ anion in interlamellar of CoNiFe-LDH [17,42]. The absorption bands below 800 cm^−1^ (755, 495, 739, 633 and 502 cm^−1^) belonged to the metal and oxygen lattice vibrations (M−O, M−O−M and O–M–O) in hydrotalcite-like lattice [17,42]. Compared with CoNiFe-LDH, FT-IR spectrum of CNTs/CoNiFe-LDH composite displayed an additional weak absorption band at 1077 cm^−1^, which was related to the vibration of C−O [31,43].

**Figure 2:**
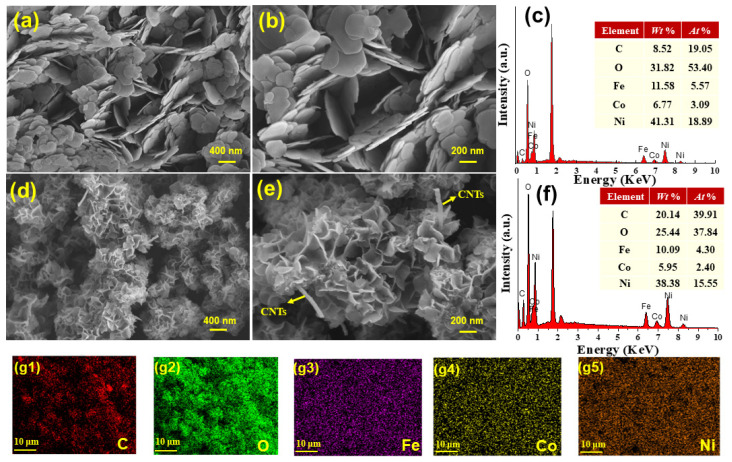
SEM images of (**a**,**b**) CoNiFe-LDH and (**d**,**e**) CNTs/CoNiFe-LDH; EDS analysis of (**c**) CoNiFe-LDH and (**f**) CNTs/CoNiFe-LDH; elemental mapping of C, O, Co, Ni and Fe in CNTs/CoNiFe-LDH (**g1**–**g5**).

**Figure 3:**
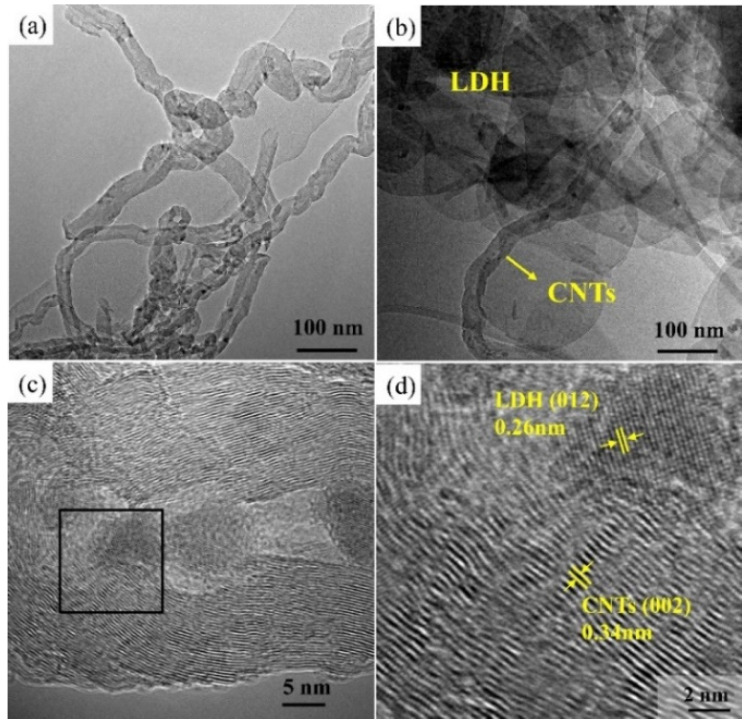
TEM images of (**a**) CNTs, (**b**) CNTs/CoNiFe-LDH. (**c**) HRTEM of CNTs/CoNiFe-LDH, (**d**) the magnified view of the selected region.

The morphologies of samples were investigated by SEM and TEM. CoNiFe-LDH displayed nanoflake-shaped structures (Figure 2a,b) and CNTs exhibited tubular-like structures (Figure 3a). As for CNTs/CoNiFe-LDH composite, LDH nanoplates were grown interlacedly on carbon nanotubes to form porous flower-like structures (Figure 2d,e and Figure 3b), which was beneficial to improve the electrochemical property of the materials. EDS analysis (Figure 2c,f) showed that the atomic percentage of C in CNTs/CoNiFe-LDH (39.91%) increased obviously compared with CoNiFe-LDH (19.05%). The elemental mapping of CNTs/CoNiFe-LDH (Figure 2(g1–g5)) showed the uniform distribution of Ni, Fe, C, O and Co elements. High-resolution TEM (HRTEM) of CNTs/CoNiFe-LDH (Figure 3c,d) displayed the lattice fringes with an interplanar distance of 0.34 and 0.26 nm, which corresponded to (002) the plane of CNTs [32,39] and (012) the plane of LDH [21,28,43,44]. These results supported the successful formation of CNTs/CoNiFe-LDH composite.

**Figure 4:**
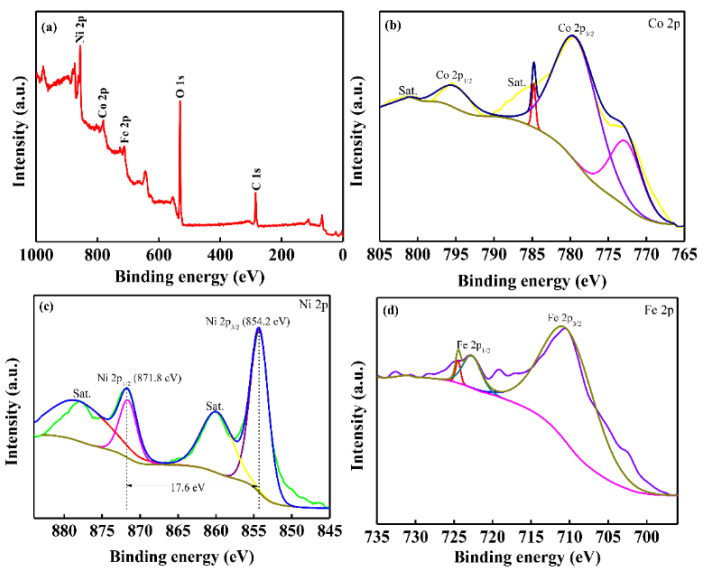
XPS survey spectrum (**a**); Co 2p (**b**); Ni 2p (**c**) and Fe 2p (**d**) of CNTs/CoNiFe-LDH.

The elemental compositions of CNTs/CoNiFe-LDH composite were further studied by XPS measurements. Ni, Co, Fe, C and O elements were detected in the survey spectrum (Figure 4a). The Co 2p spectrum (Figure 4b) exhibited two peaks at 781.5 and 797.3 eV, corresponding to Co 2p_3/2_ and Co 2p_1/2_, indicating the presence of Co^2+^ and Co^3+^ [27,28]. The Ni 2p XPS spectrum (Figure 4c) showed peaks of Ni 2p_3/2_ (856.2 eV) and Ni 2p_1/2_ (873.8 eV) with two satellites (862.2 and 879.8 eV). The spin-energy separation of 17.6 eV was characteristic of Ni^2+^ [27,28]. The Fe 2p spectrum in Figure 4d displayed two peaks of Fe 2p_3/2_ (713.0 eV) and Fe 2p_1/2_ (725.4 eV), suggesting the existence of the Fe^3+^ in the CNTs/CoNiFe-LDH composite [23,29].

**Figure 5:**
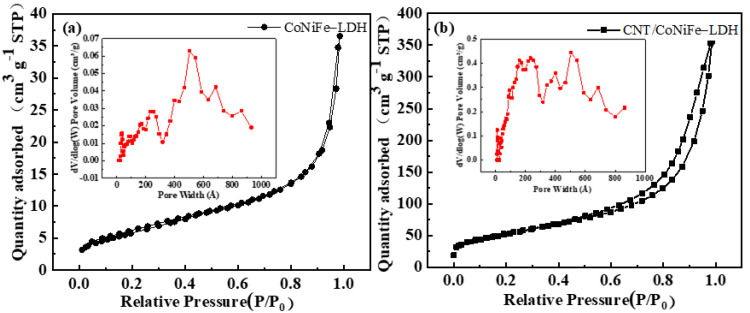
BET adsorption/desorption isotherms and the corresponding pore size distributions of (**a**) CoNiFe-LDH and (**b**) CNTs/CoNiFe-LDH.

N_2_ adsorption/desorption isotherms of CoNiFe-LDH and CNTs/CoNiFe-LDH presented type IV isotherm loops (Figure 5). The specific surface area of CNTs/CoNiFe-LDH was 189 m^2^ g^−1^, which was much larger than that of CoNiFe-LDH (21 m^2^ g^−1^). The CNTs/CoNiFe-LDH composite also exhibited a larger total pore volume (0.5176 cm^3^ g^−1^) compared with that of CoNiFe-LDH (0.0515 cm^3^ g^−1^), which was beneficial to promote charge transfer and enhance electrochemical performance [30,32]. The pore distribution of CoNiFe-LDH was mainly at 2.5~93.0 nm (inset in Figure 5a), indicating the existence of meso- and macropore structure. In comparison, CNTs/CoNiFe-LDH showed a more abundant multimodal pore distribution from 1.0~86.5 nm (inset in Figure 5b), suggesting the hierarchical porous (micro-, meso- and macropores) nanoarchitecture [28,45,46]. The result was in accordance with the SEM analysis.

TGA curves of CoNiFe-LDH and CNTs/CoNiFe-LDH contained two major descent stages (Figure S1). The weight loss at 25–170 °C and 170–400 °C can be attributed to the removal of water molecules and CO_3_^2−^, respectively [27,40]. The total weight loss of CNTs/CoNiFe-LDH and CoNiFe-LDH were 32% and 37%, indicating higher thermal stability of CNTs/CoNiFe-LDH.

**Figure 6:**
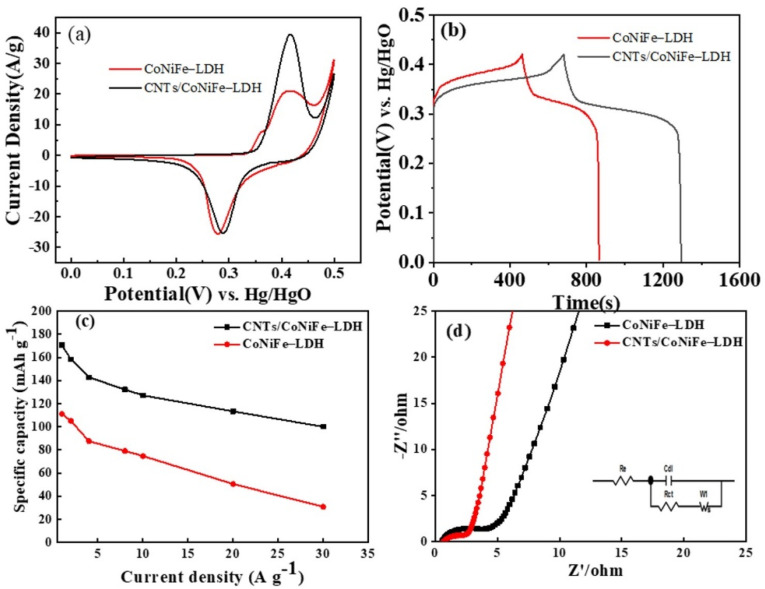
Electrochemical performance of CoNiFe-LDH and CNTs/CoNiFe-LDH: (**a**) CV curves at 5 mV s^−1^; (**b**) GCD curves at 1 A g^−1^; (**c**) specific capacity versus current density and (**d**) Nyquist plots of the electrodes of CoNiFe-LDH and CNTs/CoNiFe-LDH.

The electrochemical performance of CoNiFe-LDH and CNTs/CoNiFe-LDH was systematically studied in three-electrode configuration. CV curves (within 0–0.5 V at 5 mV s^−1^) of CoNiFe-LDH and CNTs/CoNiFe-LDH (Figure 6a) displayed a pair of redox peaks, indicating reversible redox reaction of Co^2+^ and Ni^2+^ [18,28]. Larger integral area and redox peak intensity of CNTs/CoNiFe-LDH in CV curves signified higher specific capacity. CV curves at various scan rates of CoNiFe-LDH and CNTs/CoNiFe-LDH (Figure S2a,b) demonstrated that the current of redox peaks increased and the position shifted according to the increase in scan rates, owing to the electrode polarization [30]. GCD curves (Figure 6b) of CNTs/CoNiFe-LDH showed a longer discharge time than CoNiFe-LDH, suggesting a higher specific capacity of CNTs/CoNiFe-LDH. Based on GCD curves at different current densities (Figure S3a,b), the specific capacities of CNTs/CoNiFe-LDH were 170.6, 158.3, 142.7, 132.2, 127.1, 113.3 and 100.0 mAh g^−1^ at 1, 2, 4, 8, 10, 20 and 30 A g^−1^, respectively (Figure 6c). The capacity retentions of CNTs/CoNiFe-LDH were 75% (1–10 A g^−1^), 66% (1–20 A g^−1^) and 59% (1–30 A g^−1^), which were higher than CoNiFe-LDH (67% at 10 A g^−1^ and 28% at 30 A g^−1^). Compared with CoNiFe-LDH, CNTs/CoNiFe-LDH composite had a higher capacity and superior rate retention, which could be attributed to the large surface area of CNTs/CoNiFe-LDH and the synergistic effect of carbon material and CoNiFe-LDH. The excellent rate retention of CNTs/CoNiFe-LDH composite was also superior to the reported CoNiFe-LDH [21,23,24,25] and CNTs/Ni Co LDH composites (58% from 1 to 20 A g^−1^) [32] and the Ce-NiCo-LDH/CNT electrode (67.9% from 1 to 10 A g^−1^) [31] (Table S1). Energy efficiency (η_E_) is an important parameter to evaluate electrode materials, which can be determined from the GCD curves using the relation: η_E_= E_int/D_/E_int/C_, where E_int/D_ and E_int/C_ refer to the discharge and charge energy of the electrode or device [47,48]. The energy efficiency (η_E_) of CoNiFe-LDH is found to be 72.4% at the current density of 1 A g^−1^. Compared with CoNiFe-LDH, CNTs/CoNiFe-LDH displayed improved energy efficiency (75.4%) at 1 A g^−1^, indicating the better properties of the CNTs/CoNiFe-LDH hybrid electrode. EIS analysis (Figure 6d) revealed that CNTs/CoNiFe-LDH showed larger inclination and smaller semicircle loops than CoNiFe-LDH, signifying lower internal resistance of CNTs/CoNiFe-LDH. The charge transfer resistance (Rct) values were acquired after fitting the equivalent circuit diagram (inset of Figure 6d). The fitted results of the EIS data for CoNiFe-LDH and CNTs/CoNiFe-LDH electrodes are listed in Table S2. In addition, CNTs/CoNiFe-LDH had a much smaller R_ct_ value (1.3 Ω) compared with CoNiFe-LDH (3.0 Ω), indicating a more efficient charge transfer of CNTs/CoNiFe-LDH. It could be possibly explained that high conductivity of CNTs prevented the aggregation of LDH and decreased internal resistance of the system. The excellent interfacial contact between LDH and CNTs effectively shortened the ion diffusion and migration pathways [31]. Bode modulus plots of CoNiFe-LDH and CNTs/CoNiFe-LDH are shown in Figure S4. Compared with the CoNiFe-LDH electrode, CNTs/CoNiFe-LDH displayed a much smaller impedance value resulting from the excellent electrical conductivity of CNTs.

**Figure 7:**
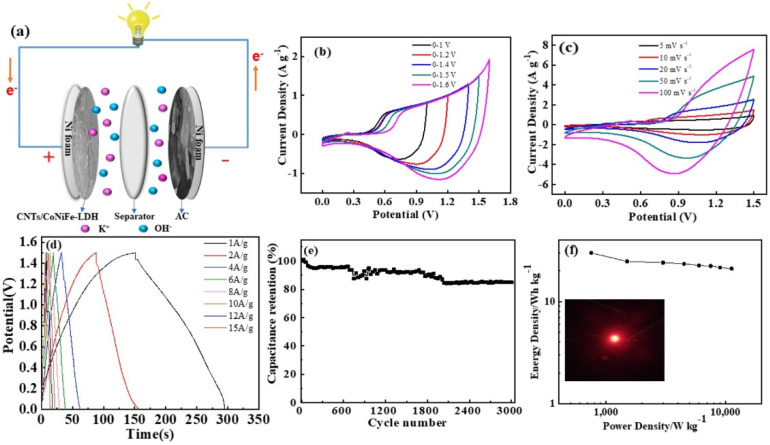
(**a**) The schematic of CNTs/CoNiFe-LDH//AC ASC; (**b**) CV curves of CNTs/CoNiFe-LDH//AC within different voltage windows at 10 mV s^−1^; (**c**) CV curves of CNTs/CoNiFe-LDH//AC at various scan rates; (**d**) GCD curves of CNTs/CoNiFe-LDH//AC at different current densities; (**e**) Cycling life of CNTs/CoNiFe-LDH//AC at 15 A g^−1^ and (**f**) Ragone plot of CNTs/CoNiFe-LDH//AC ASC.

The CNTs/CoNiFe-LDH//AC ASC was further fabricated, using CNTs/CoNiFe-LDH as a positive electrode and AC as a negative electrode (Figure 7a). It is vital to balance the charge of the positive and negative electrodes. The mass ratio of CNTs/CoNiFe-LDH and AC was calculated by $\frac{m_{+}}{m_{-}}=\frac{{\mathrm{Cs}}_{-}\times ∆V_{-}}{{\mathrm{Qs}}_{+}}$, where m_-_ and m_+_ are the masses of the negative and positive electrode, respectively. Cs- is the specific capacitance of the AC. ∆V is the potential range measured for AC. Qs+ is the specific capacity, in C/g, of the CoNiFe-LDH/CNTs composite functioning as the positive electrode. According to the above formula, the optimal mass loading ratio of active materials was m _(CNTs/CoNiFe-LDH)_:m _(*AC*)_ = 0.3. Among them, the mass of CNTs/CoNiFe-LDH was about 0.6 mg, and the mass of AC was about 1.9 mg. The electrochemical performance of AC was investigated as shown in Figure S5. The specific capacitances of AC were 182.3, 154.0, 128.8, 128.4, 128.0, 127.0, 126.0 and 124.5 F g^−1^ at 1, 2, 4, 6, 8, 10, 12 and 15 A g^−1^, respectively. CV curves of the CNTs/CoNiFe-LDH//AC ASC at potential windows from 0–1.0 V to 0–1.6 V (Figure 7b) showed that the suitable working voltage is 1.5 V with good reversibility. CV curves of CNTs/CoNiFe-LDH//AC ASC at different scan rates (within 0–1.5 V) displayed both supercapacitor-type and battery-type feature (Figure 7c). Calculated by GCD curves of CNTs/CoNiFe-LDH//AC (Figure 7d), the specific capacitances of the hybrid capacitor were 96.1, 91.9, 79.7, 78.8, 77.7, 76.8, 75.3 and 74.4 F g^−1^ at 1, 2, 4, 6, 8, 10, 12 and 15 A g^−1^, respectively (77.4%, 1–15 A g^−1^). In addition, the ASC exhibited a good cycling stability (Figure 7e). After 3000 cycles at 15 A g^−1^, the capacitance still remained over 85.0% of the initial capacitance. The capacity decay with the increase in the cycle number can be attributed to gradual collapse of the nanostructure of the active material during the charge–discharge process [27]. The Ragone plot of the CNTs/CoNiFe-LDH//AC ASC (Figure 7f) showed a high energy density of 29.9 W h kg^−1^ at the power density of 750.5 W kg^−1^. Furthermore, a red LED indicator can be powered by two CNTs/CoNiFe-LDH//AC ASC devices for more than 60 min (inset in Figure 7f), certifying its practical application in energy storage conversion devices.

## 4. Conclusions

A novel hybrid electrode material based on ternary CoNiFe-LDH (battery electrode) and CNTs (supercapacitor electrode) was firstly prepared through a one-step hydrothermal approach for hybrid supercapacitor applications. The CNTs/CoNiFe-LDH composite exhibited higher surface area, enhanced electrochemical behavior and excellent rate properties compared to CoNiFe-LDH. The CNTs/CoNiFe-LDH composite exhibited high specific capacity (170.6 mAh g^−1^ at 1 A g^−1^) and excellent rate capability (75% at 10 A g^−1^). The hybrid asymmetric supercapacitor was assembled using CNTs/CoNiFe-LDH composite as positive electrodes and activated carbon as negative electrodes. CNTs/CoNiFe-LDH//AC ASC achieved high *Cs* value (96.1 F g^−1^ at 1 A g^−1^), superior energy density (29.9 W h kg^−1^) and good cycle life (85.0% after 3000 cycles). These results demonstrated that the CNTs/CoNiFe-LDH composite exhibited high electrochemical performance, which will be a promising hybrid supercapacitor electrode.

## Data Availability

The data presented in this study is available on request from the corresponding author.

## Supplementary Materials

The following are available online at https://www.mdpi.com/article/10.3390/nano11092155/s1, Figure S1: TGA curves of CoNiFe-LDH and CNTs/CoNiFe-LDH, Figure S2. CV curves at different scan rates: (a) CoNiFe-LDH and (b) CNTs/CoNiFe-LDH, Figure S3. GCD curves at different current densities: (a) CoNiFe-LDH and (b) CNTs/CoNiFe-LDH. Figure S4. Bode modulus plots of CoNiFe-LDH and CNTs/CoNiFe-LDH, Figure S5. (a) CV curves of activated carbon at different scan rates; (b) GCD curves of activated carbon at different current densities. Table S1. Comparison of electrochemical performances of CNTs/CoNiFe-LDH in this work and other literatures, Table S2 Fitted results of the EIS data for CoNiFe-LDH and CNTs/CoNiFe-LDH electrodes.
