# Supplementary material for: Synthesis of CNTs/CoNiFe-LDH Nanocomposite with High Specific Surface Area for Asymmetric Supercapacitor
Source: Nanomaterials (Basel). 2021 Aug 24;11(9):2155. doi: 10.3390/nano11092155 (PMC8465494; doi:10.3390/nano11092155)
Supplement: Supplementary file 1 [file nanomaterials-11-02155-s001.zip › nanomaterials-1337625-supplementary.pdf]

Supplementary Material

Synthesis of CNTs/CoNiFe-LDH Nanocomposite with High Specific Surface Area for Asymmetric Supercapacitor

Jianwei Wang †, Qian Ding †, Caihui Bai †, Feifei Wang, Shiguo Sun, Yongqian Xu and Hongjuan Li *

Shaanxi Key Laboratory of Natural Products and Chemical Biology, College of Chemistry and Pharmacy, Northwest A&F University, Yangling 712100, China; wangjw19980228@163.com (J.W.); qianding@nwafu.edu.cn (Q.D.); baicaihui@nwafu.edu.cn (C.B.); wangfeifei6586@163.com (F.W.); sunsg@nwsuaf.edu.cn (S.S.); xuyq@nwsuaf.edu.cn (Y.X.)

* Correspondence: hongjuanli@nwafu.edu.cn; Tel.: +86-298-709-2226

† These authors contribute equally to this article.

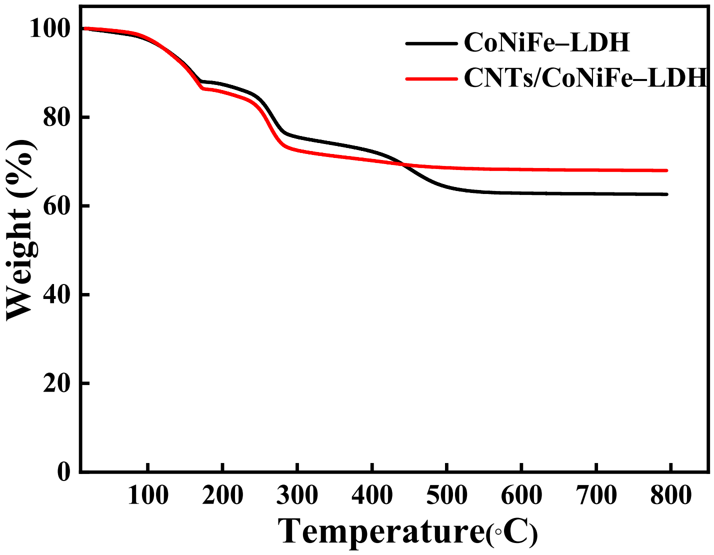

Figure S1. TGA curves of CoNiFe-LDH and CNTs/CoNiFe-LDH.

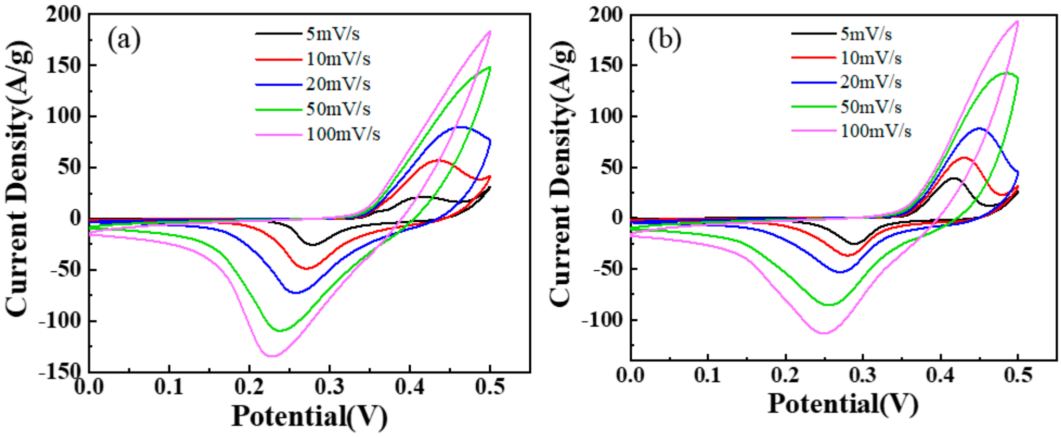

Figure S2. CV curves at different scan rates: (**a**) CoNiFe–LDH and (**b**) CNTs/CoNiFe–LDH.

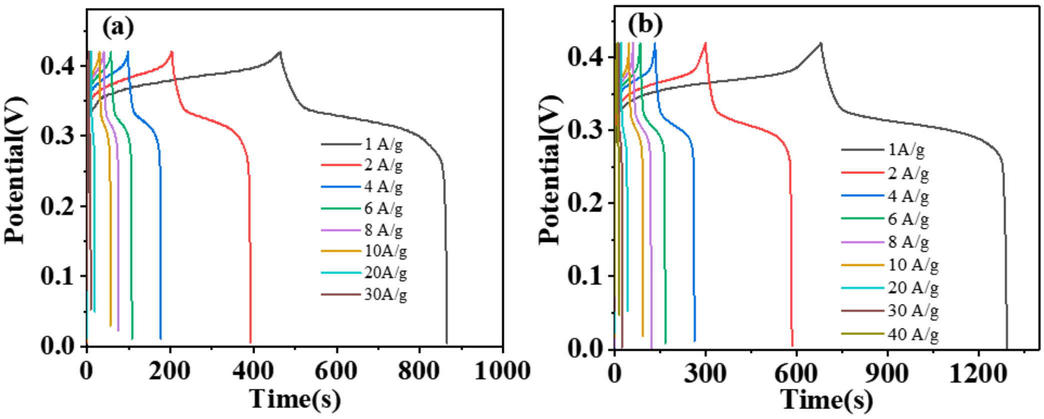

Figure S3. GCD curves at different current densities: (**a**) CoNiFe-LDH and (**b**) CNTs/CoNiFe-LDH.

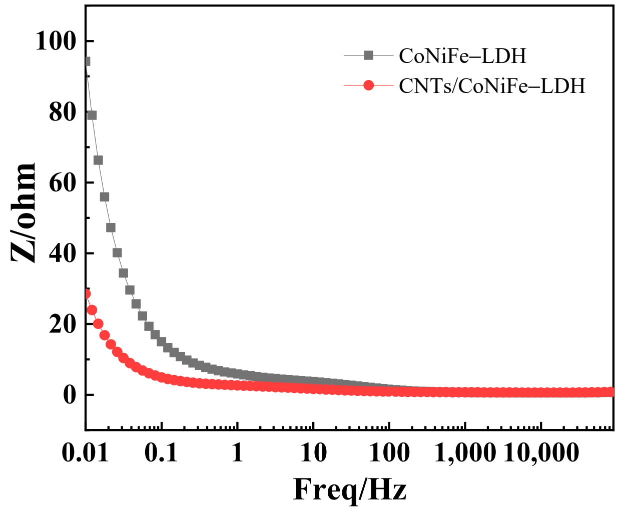

Figure S4. Bode modulus plots of CoNiFe-LDH and CNTs/CoNiFe-LDH.

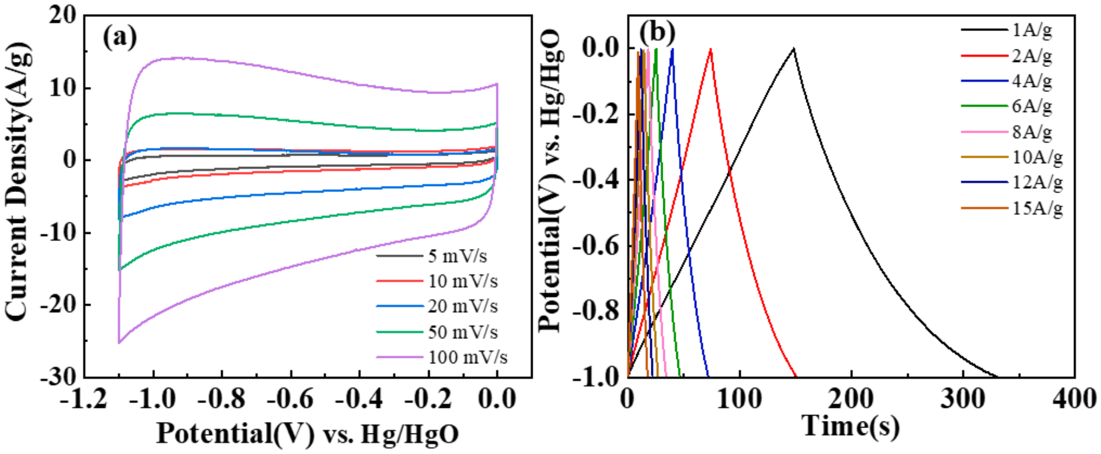

Figure S5. (**a**) CV curves of activated carbon at different scan rates; (**b**) GCD curves of activated carbon at different current densities.

Table S1. Comparison of electrochemical performances of CNTs/CoNiFe-LDH in this work and other literatures.

Electrode materials	Specific capacity	Rate capability	Device capacitance retention	Refs.
CNTs/CoNiFe–LDH	170.6 mA h g^{-1} (1 A g^{-1})<br>614.0 C g^{-1} (1 A g^{-1})	75% (1–10 A g^{-1})<br>66% (1–20 A g^{-1})	85.0% (3000)	This work
FeCoNi–LDH nanosheets	316 C g^{-1} (1 A g^{-1})	59.2% (1–10 A g^{-1})	81% (2000)	21
FeCoNi–LDH Nanocage	490 C g^{-1} (1 A g^{-1})	93% (1–20 A g^{-1})	93.1% (5000)	23
NiCoFe–LDH	541.8 C g^{-1} (1 A g^{-1})	12.8% (1–10 A g^{-1})	50.7% (3000)	24
Ce–Doped NiCo–LDH@CNT	355.7 C g^{-1} (1 A g^{-1})	68% (1–10 A g^{-1})	85.6% (9000)	31
CNTs/Ni Co LDH	732.6 C g^{-1} (1 A g^{-1})	66.6% (1–10 A g^{-1})	99.4% (10000)	32
CoNi–LDHs/SCNTF	476.2C g^{-1} (1 A g^{-1})	80.1% (1–10 A g^{-1})	81.0% (5000)	27

Table S2. Fitted results of the EIS data for CoNiFe-LDH and CNTs/CoNiFe-LDH electrodes.

Electrode	Re (Ω)	Rct (Ω)	Cdl (F)
CoNiFe–LDH	0.59823	3.016	0.0011579
CNTs/CoNiFe–LDH	0.70281	1.308	0.0067043
